# Plant-Community Vulnerability in Highly Fragmented Landscapes Is Higher in Secondary Forests Than in Old Growth Forests in the Andean–Amazonian Transition

**DOI:** 10.3390/plants11233284

**Published:** 2022-11-29

**Authors:** Carlos H. Rodríguez-León, Lilia L. Roa-Fuentes, Armando Sterling, Juan Carlos Suárez

**Affiliations:** 1 Doctorado en Ciencias Naturales y Desarrollo Sustentable, Facultad de Ciencias Agropecuarias, Universidad de la Amazonía, Florencia 180001, Colombia; 2 Programa Modelos de Funcionamiento y Sostenibilidad, Instituto Amazónico de Investigaciones Científicas SINCHI, Florencia 180001, Colombia; 3 Departamento de Ecología y Territorio, Facultad de Estudios Ambientales y Rurales, Pontifica Universidad Javeriana, Bogotá 110231, Colombia; 4 Programa de Biología, Facultad de Ciencias Básicas, Universidad de la Amazonía, Florencia 180001, Colombia; 5 Laboratorio de Ecofisiología, Centro de Investigaciones Amazónicas CIMAZ-MACAGUAL, Universidad de la Amazonía, Florencia 180001, Colombia

**Keywords:** plant community assembly, functional composition, functional diversity, functional vulnerability, successional gradient, environment filters, restoration

## Abstract

Increasing biodiversity in highly diverse plant communities can jointly increase ecosystem function and ecosystem vulnerability. This paradox requires further attention. This study analyzed the functional response of plant communities to above- and below-ground parameters along the chronosequence (degraded pastures (DP), early forests (EF), intermediate forests (IF), and old-growth forests (OF)) in two highly fragmented landscapes of the Colombian Amazon as an estimate of the level of functional vulnerability. Three sets of functional attributes were evaluated: (i) functional composition based on the community-weighted mean (CWM) of five traits; (ii) functional diversity based on the multi-trait indices and functional dispersion (FDis) of each individual trait; and (iii) the functional vulnerability at the community-level and species-level. The individual traits did not show a clear pattern along the chronosequence. However, the trend indicated an increase in the values of resource conservation traits with the age of abandonment. The functional response of the community did not vary between landscapes. Between DP and OF, there was a significant increase in functional diversity and a decrease in functional redundancy, which increased community-level vulnerability. Consequently, the more vulnerable species were observed in the IF and OF plots. In addition, a decrease in environmental parameters, such as penetration resistance, bulk density and Ca content, and an increase in slope, precipitation, electric conductivity, pH, clay, organic material, and P and N contents increased the vulnerability. We elucidated the need for secondary forest management in terms of conservation and restoration to maintain the capacity to respond to changing environmental conditions in highly fragmented landscapes in the Andean–Amazonian transition.

## 1. Introduction

The study of ecosystem functional complexity shows the relationships between biodiversity, abiotic filters, and ecosystem functions when taxonomic and phylogenetic classifications alone cannot [1,2,3]. For instance, plant species communities display a range of trait variation that determines the community response to abiotic factors (above- and below-ground factors), persistence, and the species abundance ratio. Abiotic conditions varied from the local to the regional scale, assigning a differential filter to the community assembly. Land use history results in various contrasting environmental conditions, which can determine the trajectory of plant recovery. Cattle pasture abandonment provides new areas for natural regeneration; however, soil properties after use tend to have reduced soil organic matter, nitrogen, phosphorus, and aggregate stability [4], which could determine which species persists in these sites [5]. In addition, shifts in community composition across the secondary succession can drive changes in ecosystem properties, mediated by species traits [6,7]. The relationship between environmental conditions and plant species composition works in both directions. The evidence suggests coordination between plant functional diversity and succession [8], which is driven by variations in physical environments [9,10]. However, changes in environmental conditions affect various species differently, depending on the values of the functional traits.

Tropical humid forests in the Amazonian basin are strongly impacted by land use changes, including cattle pasture use [11], plant community composition and functional diversity, which are expected to be affected by combined environmental conditions and the regional pool of species. Consequently, the natural regeneration across the chronosequence is an opportunity for understanding the vulnerability of recovered forests and tracing signals of secondary forests.

Vulnerability is an increasing function of the species functional relevance and its extinction probability [12]. The vulnerability across the chronosequence will tend to increase in a highly diverse community when rare species that are functionally dissimilar (i.e., species with leas common trait values) tend to increase [12,13]. Likewise, a larger number of functionally dissimilar species in a community (i.e., functional uniqueness) means a lower probability of maintaining the properties of an ecosystem. This is because the disappearance of one or more of those species will affect that ecosystem significantly because the remaining species cannot compensate for it (i.e., low functional redundancy) [3,12,14].

In addition, it has been well documented that an increase in functional diversity and trait variance is associated with an increase in ecosystem function since biodiversity increases the resistance and resilience of an ecosystem function to biodiversity loss [3,15,16]. However, other studies have shown that an increase in biodiversity may increase the functional vulnerability of the community since a greater variance in the functional space associated with functionally dissimilar and sensitive species would reduce the compensatory capacity of the ecosystem [12,13,17]. In this sense, the paradox of how biodiversity can jointly increase the function and vulnerability of ecosystems needs to be addressed with more empirical evidence [17].

On the other hand, since a new set of environmental conditions is expected to be found during the chronosequence, the species present in this set of conditions could maintain a low abundance, exposing the community to conditions of vulnerability. If that were the case, the secondary forests, which are seen as an opportunity to recover biodiversity and other functions, would be configuring an alternative state of low stability in an unpredictable trajectory. However, the empirical evidence remains inconclusive.

In the present study, the functional response of plant communities to above- and below-ground parameters was analyzed along the chronosequence in two highly fragmented tropical humid forest landscapes. The research aimed to predict the vegetation functional dynamics with two questions:

1. How do the effects (i.e., magnitude and direction) of abiotic environmental filters on the taxonomic and functional diversity of the plant community change along the gradient of soil abandonment (i.e., chronosequence); and 2. Does the changing trend lead the plant community to conditions of greater functional vulnerability? Additionally, this research quantified the synergistic effect of topographic and climatic factors and soil properties on the diversity of species and the functional attributes of the plant community to determine whether abiotic filtration is the dominant assembly process in the secondary forests of the tropical humid forest or, on the contrary, whether the current ensemble is due to stochastic processes. Following the generated patterns, a greater functional similarity is expected between the species that coexist in the early stages of succession. Finally, plants’ taxonomic and functional diversity is expected to increase jointly with vulnerability from early to late successional stages, along with increasing environmental divergence.

## 2. Materials and Methods

### 2.1. Study Area

The study area was in the municipalities of San José del Fragua (1°19′52″ N; 75°58′28″ W), Belén de los Andaquíes (1°24′59.1″ N; 75°52′21.2″ W), Morelia (1°29′09″ N; 75°43′28″ W) and Florencia (1°36′50″ N; 75°36′46″ W) in Caquetá state in two highly fragmented landscapes (hill and mountain) in the Andean–Amazonian transition ( Supplementary Figure S1). The hill landscape corresponded to an undulated topography with areas of mainly livestock use and some forest relicts, while the mountain landscape was characterized by higher slopes and a mosaic of natural forests, pastures, and agricultural land as described in previous studies [18,19]. The study area had a humid-warm climate characterized by a monomodal regime with an average annual precipitation of 3376 mm and an average temperature of 25.04 °C. The study zone had Oxisols and Ultisols soils with low fertility and aeration, acids (pH < 6), high aluminum saturation (>60%), and a clayey texture [20].

### 2.2. Field Sampling and Species Selection

As part of a study relating to forest structure and diversity across the chronosequence in mountainous and hilly landscapes, 33 plots (50 × 50 m) were selected that encompassed the range of landscape succession states from degraded pasture (DP, <3 years), early forest (EF, 10 to 20 years), intermediate forest (IF, 25–40 years), and old-growth or mature forest (OF, >90 years), as described in previous studies [18,19]. In each landscape, 14 and 19 plots (hill and mountain, respectively) were established as follows: (i) two plots in DP; (ii) three plots in EF; (iii) six plots in IF, and (iv) three plots on OF in the hills. Three plots in DP, three plots in EF, six plots in IF, and seven plots in OF in the mountain landscape. Stand age was assigned based on the local expert knowledge and specific site history in the area for at least 40 years. Plant traits sampling was restricted to individuals with a diameter at breast height (DBH) > 10 cm. Species with at least 55% of the cumulative importance value index (I.V.I) in each chronosequence were selected. Of a total of 541 fully determinate species, 213 were selected.

### 2.3. Traits Sampling

The measurement of species composition and growth habits was undertaken from February to December 2017. All plots and trees were marked in the field. In each plot, all trees with a diameter at breast height greater than 1 cm (DBH) were counted and identified to the species level, and the diameter at DBH was measured. All specimens were processed, identified, and deposited in “Herbario Amazónico Colombiano—COAH” of the Sinchi Institute in Bogotá D.C, Colombia. We sampled almost one individual per plot as was suggested for species-rich plant communities [21]. The individual was randomly selected from the known individuals within 0.25 ha plots to each chronosequence. We collected five mature, healthy, and completely expanded leaves from sun-exposed branches of every individual. We mixed leaves within each tree and stored them in water-saturated conditions [22]. Foliar and stem traits were measured following the standardized methods in Perez-Harguindeguy et al. [23] handbook. We measured leaf area (LA, mm^2^), leaf thickness (LTh, mm), leaf dry matter content (LDMC, mg dry mass g^−1^), specific leaf area (SLA, mm^2^ mg^−1^) and wood density (WD, g cm^−3^).

### 2.4. Measurements of Functional Composition, Diversity and Vulnerability

The functional composition was based on community-weighted mean (CWM) of trait values according to Garnier et al. [24]. The functional diversity was quantified with different community-level multi-trait-based indices [2,25]: (1) functional richness (FRic); (2) functional evenness (FEve); and (3) functional divergence (FDiv). The functional diversity was also estimated according to Laliberté et al. [16] as follows: functional dispersion (FDis) was calculated first in a multidimensional functional space, and then for each individual trait, according to Hu et al. [26]. All functional traits values were standardized to 0 mean and 1 variance before quantifying functional diversity.

Finally, various functional-vulnerability-related aspects were analyzed according to Ricotta et al. [12]. Thus, five metrics were calculated: (1) species richness (S); (2) Gini–Simpson diversity index (D) [27]; (3) Rao’s quadratic diversity (Q) as proxy of community-level functional vulnerability (V) [28]; (4) community-level functional redundancy (R) [12]; and (5) species-level functional vulnerability (Vi) [12].

### 2.5. Environmental Parameters

In this research, 28 above- and below-ground environmental parameters were evaluated in each plot as follows:

(i) Five above-ground variables (i.e., topographic and climatic parameters): slope (%), elevation (m), temperature (°C), precipitation (mm year^−1^) and environmental stress, according to previous studies [19]. The slope was measured with a laser hypsometer (Forestry Pro II), while the elevation was recorded with a digital altimeter (GPSMAP 64CSX). Temperature and precipitation were obtained from Worldclim dataset [29]. Environmental stress was defined according to Chave et al. [30].

(ii) Twenty-three below-ground variables as described in previous studies [18,19]: (a) soil physical properties: structural stability index (SI) (%) [31], texture (sand, clay, and silt) (%), total porosity (%), penetration resistance (MPa), bulk density (g cm^−3^) and soil moisture (%) [32]; (b) soil chemistry: soil organic carbon content (SOC) (%) [33], available phosphorus (P) (mg kg^−1^) [34], pH, electric conductivity (EC) (dS m^−1^), cation exchange capacity (CEC) (meq 100 g^−1^), exchangeable acidity (EA) (mg kg^−1^), total nitrogen (N) (%), calcium (Ca) (mg kg^−1^), magnesium (Mg) (mg kg^−1^) and potassium (K) (mg kg^−1^) [32]; (c) soil aggregate morphology: physical macroaggregates, root macroaggregates, biogenic macroaggregates, non-macroaggregates and organic material (leaves, roots and vegetables fragments) [35].

### 2.6. Data Analysis

To test for differences in the functional variables between the fixed effects of chronosequence (DP, EF, IF, and OF), landscape (hill and mountain) and the interaction between them, a general linear model (GLM) with two-way ANOVA was fitted for each variable, using the lme function from R package nlme [36] in R language and the interface in InfoStat software v. 2020 [37]. The assumptions of normality and homocedasticity were evaluated using exploratory residuals analysis. Differences between means in the fixed effects were analyzed with Fisher’s LSD post hoc test at a significance of *α* = 0.05. A Principal Component Analysis (PCA) was carried out, applying a Monte Carlo test (999 permutations) to evaluate the significance (*α* = 0.05) in the overall effect of main factors on the CWM traits and soil properties, using the dudi.pca and randtest functions from R package ade4 [38]. The effects of the environmental parameters on CWM traits were evaluated with a Redundancy Analysis (RDA) using the function rda from R package vegan [39]. A Pearson correlation analysis was performed to analyze the level of association between all the variables. The visualization of the Pearson correlation matrix was carried out with the corrplot function from R package corrplot [40]. To test the relationships between the functional indices and environmental parameters, linear regression models were fitted with permutation tests (999 permutations) using the lmp function from R package lmPerm [41]. Finally, based on the fitted linear regression models, stepwise selection, and previous studies [26,42,43,44,45], structural equation models (SEMs) were used to evaluate the above- and below-ground environmental effects on community taxonomic and functional diversity. To examine the fitness of the SEMs, two statistics, global goodness-of-fit (i.e., Fisher’s C test) [26,46] and Akaike’s Information Criterion (AIC) [45,47], were used. The SEM analysis was conducted with the psem function from R package piecewiseSEM [48]. The analyses in R language were performed in R v. 4.0.3 [49] using the interface in RStudio v.1.3.1093 [50].

## 3. Results

### 3.1. Functional Composition

**Figure 1:**
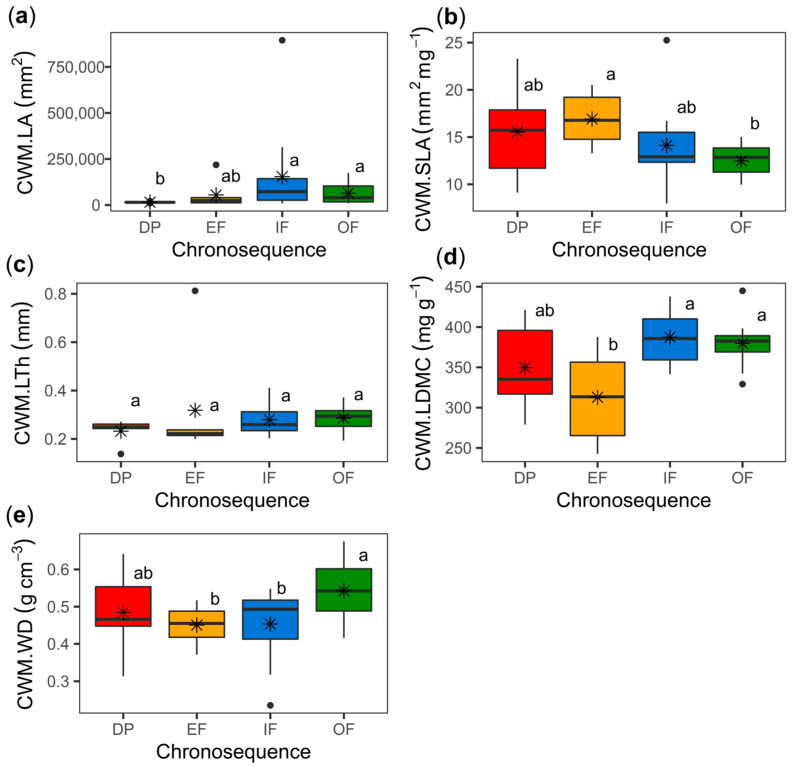
Community-weighted means (CWMs) of plant traits for the different successional categories (DP, degraded pasture; EF, early forest; IF, intermediate forest; OF, old-growth forest or mature forest); (**a**) LA, leaf area; (**b**) SLA, specific leaf area; (**c**) LTh, leaf thickness; (**d**) LDMC, leaf dry matter content; (**e**) WD, wood density. Asterisk (*) represent mean values. The upper and lower whiskers represent the maximum and minimum values established at the third (+1.5 IQR) and first quartile position (−1.5 IQR), respectively. For each trait, values between successional categories followed by the same letter do not differ statistically according to Fisher’s LSD post hoc test (*p* < 0.05).

Significant changes in the CWMs of the plant traits were observed along the successional gradient, except for CWM.LTh (Table 1). However, no significant changes in traits were evidenced between landscape units or in the interaction of the successional stage with the landscape. Traits such as LA, LDMC and WD, tended to increase along the successional gradient although the CWMs of LDMC and WD for OF and IF were not significantly different from DP (*p* > 0.05) (Figure 1). The SLA tended to decrease as succession progressed although SLA was not significantly different between DP and OF (*p* > 0.05), contrary to EF vs. OF (*p* < 0.05). Higher differences between EF and OF were observed in SLA, LDMC and WD (Figure 1).

**Figure 2:**
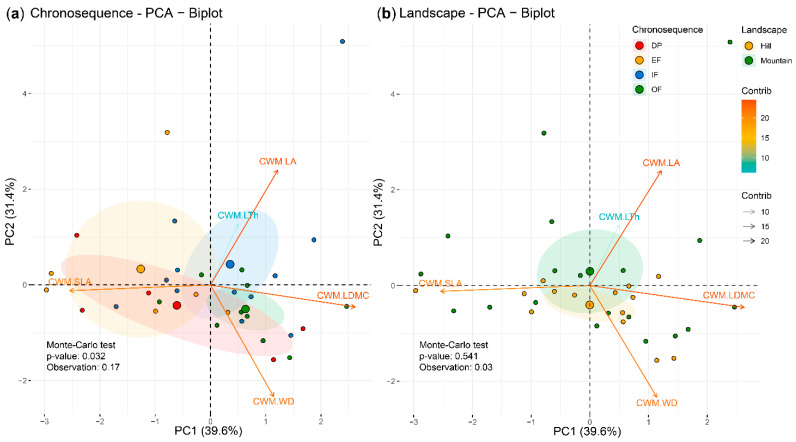
Principal component analysis (PCA) with the community-weighted means (CWMs) of plant traits and the sampling plots projected on the ordination plane PC1/PC2. The color of the vectors indicates the contribution of the variables to the PCs; 95% confidence ellipses. (**a**,**b**) Sampling plots grouped by chronosequence (DP, degraded pasture; EF, early forest; IF, intermediate forest; OF, old-growth forest or mature forest) and landscape (hill and mountain), respectively.

The relationships between the CWMs of the traits, the successional categories and landscapes were evaluated with PCA (Figure 2). The first two principal components explained 71.0% of the variance, with the CWMs of the traits grouped into clusters that were clearly defined according to the successional categories (Figure 2a) (*p* < 0.05; 17% of explained variance) but not for the landscape units (Figure 2b). Overall, greater values of LA, LTh and LDMC were strongly associated with plots with a higher abandonment age (OF and IF), while SLA showed a higher relationship with plots with a lower age (DP and EF). The WD was more associated with OF and DP plots (Figure 2a). At the landscape level (Figure 2b), LA and LTh had higher association with the mountainous landscape, while WD did so with the hilly landscape.

### 3.2. Functional Diversity

**Figure 3:**
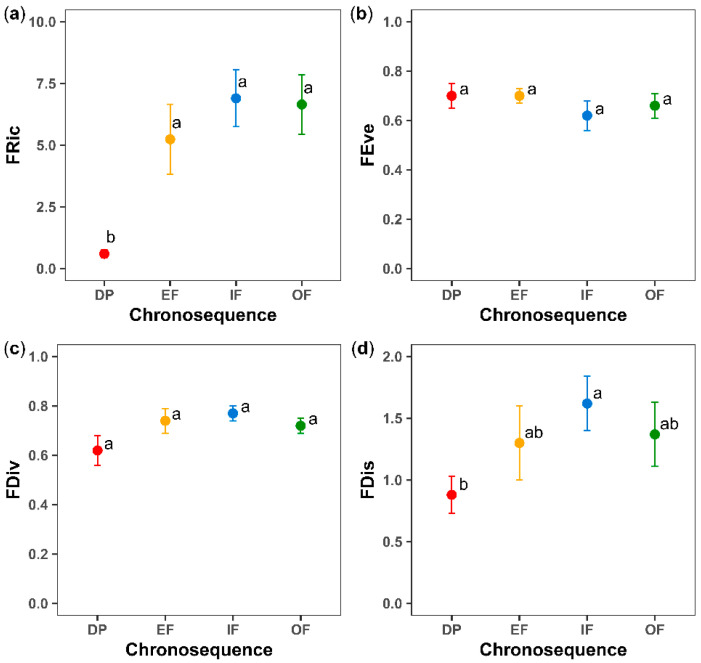
Means ± SE for: (**a**) functional richness (FRic), (**b**) functional evenness (FEve), (**c**) functional divergence (FDiv), and (**d**) functional dispersion (FDis) for the different successional categories (DP, degraded pasture; EF, early forest; IF, intermediate forest; OF, old-growth forest or mature forest). For each index, values between successional categories followed by the same letter do not differ statistically according to Fisher’s LSD post hoc test (*p* < 0.05).

The results of two-way ANOVA showed a significant effect of the chronosequence on FRic, FDis and FDis.LA, landscape on FDis.WD, and interaction of the chronosequence with the landscape on FEve (Table 1). According to Fisher’s LSD test, DP showed significantly lower mean values of FRic than other successional categories (*p* < 0.05; Figure 3a) and lower FDis values than IF (*p* < 0.05; Figure 3d). In contrast, in the mountainous landscape, DP had higher FEve values than IF and OF, while in the hilly landscape, there were no significant changes along the successional gradient.

For the FDis of the individual traits, no significant changes were evidenced along the chronosequence (*p* > 0.05), except for FDis.LA, which decreased significantly in DP when compared to the other successional stages.

### 3.3. Functional-Vulnerability

The chronosequence had significant effects on functional-vulnerability-related indices (S, Q, R and Vi; all, *p* < 0.05), except for D (Table 1). The landscape did not have significant effects on the functional vulnerability (*p* > 0.05). In addition, the interaction of the chronosequence with the landscape only had a significant influence on Vi.

**Figure 4:**
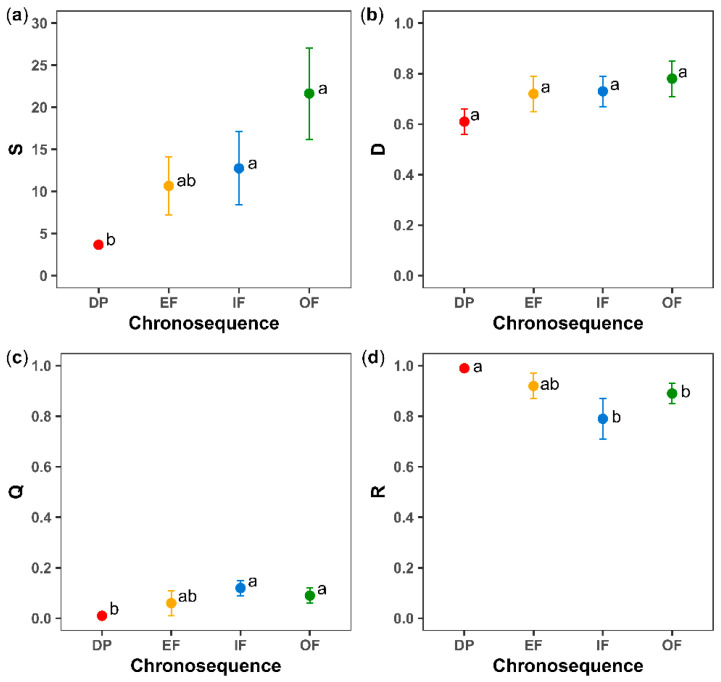
Means ± SE for: (**a**) species richness (S), (**b**) Gini–Simpson index (D), (**c**) Rao quadratic diversity (Q, measure of functional diversity, as well as average community-level vulnerability), and (**d**) community-level functional redundancy (R) for the different successional categories (DP, degraded pasture; EF, early forest; IF, intermediate forest; OF, old-growth forest or mature forest). For each index, values between successional categories followed by the same letter do not differ statistically according to Fisher’s LSD post hoc test (*p* < 0.05).

Overall, the S, D and Q indices tended to increase along the successional gradient (Figure 4a–c), while R tended to decrease as the succession progressed (Figure 4d). Thus, S an Q were significantly higher in the IF and OF categories than in DP (*p* < 0.05), contrary to that observed in R.

**Figure 5:**
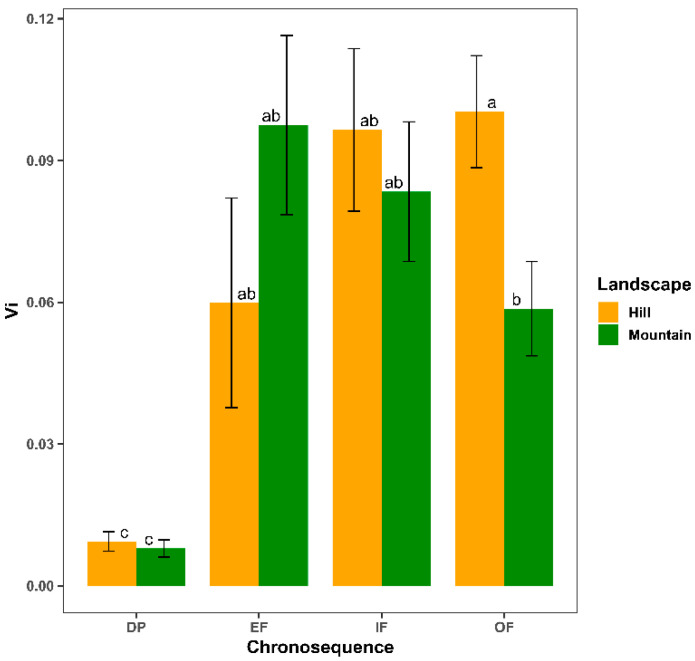
Means ± SE for species-level vulnerability (Vi) for the different successional categories (DP, degraded pasture; EF, early forest; IF, intermediate forest; OF, old-growth forest or mature forest) compared in the two landscape types (hill and mountain). Values between landscapes within each successional category followed by the same letter do not differ statistically according to Fisher’s LSD post hoc test (*p* < 0.05).

On the other hand, in both landscapes, a higher species-level vulnerability (Vi) was evidenced in the EF, IF and OF categories than in DP (*p* < 0.05; Figure 5. OF had a significantly higher Vi in the hill than in the mountain (*p* < 0.05).

In relation to the vulnerability of the species reported in each plot, the more vulnerable species (Vi > 0.5) were: *Socratea exorrhiza* (Mart.) H.Wendl. in IF in hill (Vi = 0.91), *Virola elongata* (Benth.) Warb. in IF in the mountain (Vi = 0.83), *Tapirira guianensis* Aubl. in IF in the mountain (Vi = 0.83), *Oenocarpus bataua* Mart. in OF in both the mountain (Vi = 0.74; Vi = 0.63) and the hill (Vi = 0.72; Vi = 0.68) and *Alsophila cuspidata* (Kunze) D.S.Conant in EF in the mountain (Vi = 0.65) ( Supplementary Table S1).

### 3.4. Environmental Filters and Their Relationships with Functional Attributes

The PCA showed that the successional categories and landscape types were grouped into significantly defined clusters, depending on above- and below-ground environmental parameters (Supplementary Figure S2). Overall, the soils with higher values of bulk density, penetration resistance and root macroaggregates were strongly associated with the DP plots, while the IF and EF plots were mainly related to the hill landscape and had higher pH, clay, CEC, EA and physicogenic macroaggregates values. Furthermore, higher values of precipitation and temperature were reported in the IF and EF plots. The OF plots, mainly in the mountain, had higher elevation and slope values and soils with greater values of EC, Si, organic material and non-macroaggregates.

The RDA of the CWMs of the traits, constrained by above- and below-ground environmental parameters (constrained inertia = 96.62%), separated the DP plots from OF and IF plots along the first ordination axis (RDA1 = 95.8%) (Supplementary Figure S3). However, differences between the mountain and hill centroids were not found. The penetration resistance, root macroaggregates and environmental stress significantly explained more of the variation between the plots (all, *p* < 0.05) as well as the CWMs of five traits studied (all, *p* < 0.01).

Based on Correlation Pearson’s test (Supplementary Figure S4), the higher significant correlations between the functional attributes and environmental parameters, corresponded to: CWM.WD with organic material (*r* = 0.55, *p* = 0.001); R with penetration resistance (*r* = 0.48, *p* = 0.004); and CWM.LA, FDis, FDis.LA and Q with penetration resistance (all, *r* < −0.40, *p* < 0.05).

The results of the regression analysis (Supplementary Figures S5–S7) showed that: the FDis, FDis.LA, FDis.WD and Q indices were positively related to organic material, while FDis, FDis.LA and Q were negatively related to penetration resistance. FRic and FDis.WD were positively related to slope; FRic and FDis.SLA were positively related to Ca; FEve was positively related to EA; and S and FDis.WD were positively related to EC. Other environmental parameters that positively or negatively influenced some of the functional indices were pH, CEC, Mg, P, silt, precipitation and temperature.

**Figure 6:**
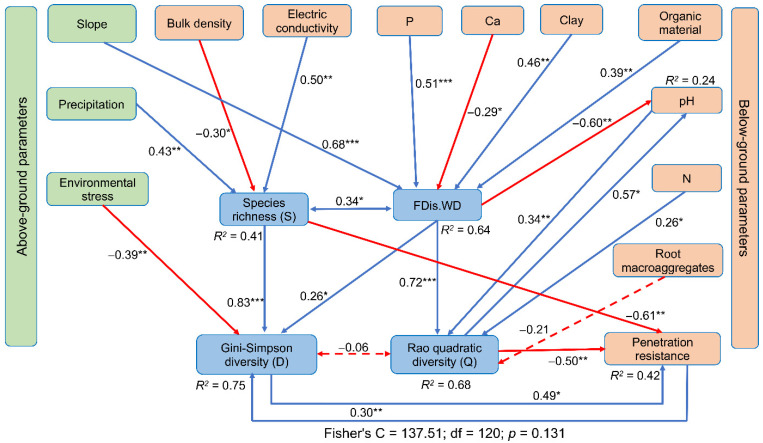
Structural equation model (SEM) selected for the effects of above- and below-ground environmental parameters (green and orange rectangles, respectively) on taxonomic (S and D) and functional (FDis.WD and Q) diversity (both, blue rectangles). Functional dispersion of wood density (FDis.WD). Blue solid arrows represent significant and positive effects, while red solid arrows indicate significant and negative effects. Dashed arrows indicate no significant effects. Blue double-headed solid arrows represents significant and positive residual covariance, while red double-headed dashed arrows denote no significant residual covariance. Values adjacent to the arrows correspond to the standardized coefficients. Individual *R^2^* indicate the proportion of variance explained for response variable. Global goodness-of-fit test based on Fisher’s C statistic. *, **, and *** indicate *p* < 0.05, *p* < 0.01, and *p* < 0.001, respectively.

Finally, the SEM results showed a relationship between taxonomic and functional diversity, with above-ground environmental parameters (i.e., climatic and topographic characteristics) (Figure 6). That is, when precipitation increased, the species richness (S) became higher; consequently, the greater S had a positive effect on the taxonomic diversity (D). In contrast, the environmental stress had a negative effect on D. Furthermore, an increase in the slope had a positive effect on the functional dispersion of wood density (FDis.WD), which in turn had a positive effect on the functional diversity (Q) (i.e., on community-level functional vulnerability).

For the below-ground parameters (i.e., soil properties), the SEM showed that bulk density and electric conductivity (CE) had a negative and positive effect on S, respectively, which led to an increase in D. Furthermore, an increase in the penetration resistance increased D. On the other hand, when the P, clay and organic material contents increased, the FDis.WD became higher; consequently, the higher FDis.WD had a positive effect on Q. In addition, when the pH and N values increased, Q became higher.

The SEM also confirmed that S and Q had a negative effect on the penetration resistance, contrary to that observed with D. In addition, the pH responded negatively to the effect of FDis.WD and positively to Q.

## 4. Discussion

As seen in previous research, our results suggest an increase in the values of conservative strategy with the age of abandoned land use [8,26], providing the community with resistance to adverse environmental conditions. However, the weakness of such a pattern indicated that old-growth forests in secondary succession might be in a state of transition to mature forests. The multivariate analysis confirmed the findings using individual traits. Forests with a higher age of abandonment (OF and IF) are strongly related through higher values of LA, LDMC and WD in contrast to forests with a lower age of abandonment (DP and EF). The functional response of the plant community was consistent across of the landscapes, which showed a regional pattern rather than a landscape scale.

The lack of variation in the functional richness after ten years of abandonment contrasted with the increase in species richness reported for the same stands in Rodríguez-León et al. [19] and patterns reported in other studies along the chronosequence [7,8,51]. This pattern indicated that adding new species to the plant community is not generating niche differentiation [3], and the plant species are being selected in a range of similar trait values to tolerate the environment conditions. In concordance, low variation in functional richness in forests between 10 and >90 years indicates a tendency towards homogenization in the values in the regional pool of tree species, which may show signs of vulnerability in plant communities before changing conditions. The increase in the community vulnerability after ten years of abandonment is explained by the richness of singularity species (i.e., species that are functionally distinct) throughout the chronosequence. A greater number of singularity and low-abundance species (rare species) has been previously reported in old forests [13,52,53]. Therefore, common species are showing their ability, given the value of their traits, to adapt to current conditions through greater abundance. However, some studies have suggested that functional traits with high effects on ecosystem functioning in sensitive species could not be mitigated by other species, and, consequently, the ecosystem would lose relevant functions [12,17]. Additionally, between DP and OF, there was a significant increase in functional diversity and a decrease in functional redundancy, which increased community-level vulnerability, a pattern reported in other studies [12,13,17].

Our results agree with Mouillot et al. [13], who stated the need for “the conservation of rare species, even in highly diverse ecosystems”. Rare species increase the functions of the community and, in turn, provide stability in changing conditions [13] without increasing functional space [54]. This, contrasts with the “insurance hypothesis”, in the sense that a greater functional space (i.e., functional richness), increases variation in species responses to variable conditions and disturbances [3]. In addition, this study confirmed that more unique species were more vulnerable (Vi > 0.5), and, therefore, they mainly affect the vulnerability of the ecosystem due to a lack of redundancy community [12,13,17,27].

Regardless of the age of abandonment, the functional richness increased significantly with the slope and the calcium content of the soil; that is, these factors can determine trends of change in the functional space of plant communities. The evenness and functional divergence of the plant community were not sensitive to changes along the indirect gradient of the chronosequence. In contrast, these responded to specific environmental conditions. FEve is the most sensitive attribute of functional diversity. An increase in environmental stress values (i.e., temperature, precipitation, and water deficit [19] will increase the FEve affecting the distribution of the species abundance in occupied niche space [55]. Such adjustment can be achieved by increasing the abundance of singular species or reducing functionally common species, moving the plant community to increase productivity [55] without changing the functional space. Low FDis in degraded pastures suggests a higher similarity in the plant community assemblage; the individual similarity decreases with resistance to soil penetration while increasing with the amount of organic matter, which highlights the adverse effect of compaction produced by the activities of use, mainly livestock. In contrast, higher values of FDis in forests older than 10 years could be a shift from environmental filtering to stronger competition interactions as forests get older [8].

The forests with longer abandonment (IF and OF) showed greater individual dissimilarity in the community assembly, and this often increased the resistance and resilience of ecosystem function [15]; however, a highly diverse community is typically composed of many rare and functionally dissimilar species [12] or more speciose communities that include functionally distinct and sensitive species that can increase community vulnerability [17]. Our results showed a lower functional redundancy in IF and OF, and consequently, an increase both in the community-level functional vulnerability and species-level vulnerability, especially in the hill landscape for the latter.

Understanding the synergistic effect of above-ground and below-ground factors on quantitative community attributes can help understand species assembly mechanisms through natural regeneration. Pinho et al. [56] suggest that tree assemblages in regenerating tropical forests may be a process determined by soil fertility, causing a functional convergence towards a more conservative use of resources. The results showed that the increase in soil bulk density can negatively affect species richness (S), while the increase in S would reduce the resistance to penetration. Additionally, resistance to penetration was negatively affected by the increase in functional diversity (Q). This relationship pattern emphasized the need to restore the components of taxonomic and functional diversity to reverse the conditions of soil penetrability.

The effect of cattle trampling on physical soil conditions has been widely reported [4,18,57,58]. The negative impact on apparent density and resistance to penetration is associated with the breakdown of aggregates, less aeration, and reduction in water retention capacity [10]. The latter can generate a selective effect on plant species that can persist or be excluded depending on rooting ability, nutrient absorption, and breathability [55,59].

Soil organic matter, P, N, and Ca had a positive effect on community dispersal using FDis.WD. Similarly, Hu et al. [26] reported a positive effect of soil N and pH on multi-trait FDis. Higher soil fertility favors increased species diversity with a higher wood density because species in the community have different abundances; less abundant species contribute higher values to the functional dispersion of WD. Similarly, a higher FDis.WD value is related to high Q values for this case (i.e., a higher average community-level functional vulnerability), where FDis is calculated univariate.

Finally, the results showed that conditions of greater environmental stress tend to reduce the diversity of species (D), limiting the number of species (S) that may form a community. This is in agreement with other studies, suggesting that plant species will respond to climatic changes with adaptation difficulties, consequently reducing species diversity [59,60].

## 5. Conclusions

This study confirmed that taxonomic and functional diversity changes occurred throughout the chronosequence, depending on the type of environmental condition and the magnitude of the environmental change. There was no further increase in functional richness between the early and old-growth forests despite the increase in alpha diversity, but there were significant changes in functional diversity between the degraded pastures and the other successional stages jointly with a decrease in functionally redundant species, thus increasing the community-level functional vulnerability, with a tendency toward older forests (i.e., intermediate and old-growth forests). Furthermore, results show a deterministic pattern dominating the plant community assembly with a response to the environmental conditions. Then, the decrease in some soil properties (i.e., penetration resistance, bulk density and Ca content), and the increase in slope, precipitation, and soil properties such as EC, pH, clay, organic material and P and N contents, are abiotic filters acting synergically to move the plant community to a more vulnerable state, increasing the number of low-abundance species with extreme trait values or functionally distinct and sensitive species (i.e., higher functional uniqueness). In concordance with our expectations, the increase in plants’ taxonomic and functional diversity is shown to increase vulnerability in the plant community from early to late successional stages, along with increasing environmental divergence. The results are empiric evidence of the “diversity paradox” proposed by Heilpern et al. [17].

We emphasized the need for secondary forest management in conservation and ecological restoration of relevant species to maintain the capacity of the plant community to respond to changing environmental conditions in highly fragmented landscapes in the Andean–Amazonian transition.

## Figures and Tables

**Table 1:** Effects of chronosequence and landscape on different plant functional attributes along succession in the Colombian Andean–Amazonian transition.

Factor	Chronosequence			Landscape			Chronosequence × Landscape		
Variables	*df*	*F*	*p*	*df*	*F*	*p*	*Df*	*F*	*p*
CWM.LA	3	3.37	0.034	1	2.09	0.161	3	0.79	0.513
CWM.LTh	3	1.03	0.397	1	0.99	0.330	3	0.91	0.448
CWM.SLA	3	3.27	0.038	1	0.25	0.625	3	0.15	0.928
CWM.LDMC	3	3.03	0.046	1	0.04	0.845	3	0.19	0.900
CWM.WD	3	3.09	0.045	1	3.21	0.085	3	0.12	0.948
FDis.LA	3	5.87	0.004	1	1.10	0.305	3	0.73	0.544
FDis.LTh	3	0.66	0.583	1	1.40	0.248	3	0.67	0.577
FDis.SLA	3	0.43	0.733	1	0.21	0.651	3	0.81	0.501
FDis.LDMC	3	0.22	0.882	1	0.79	0.384	3	0.16	0.923
FDis.WD	3	1.43	0.257	1	5.72	0.025	3	0.45	0.720
FRic	3	4.41	0.013	1	0.30	0.588	3	0.56	0.644
FEve	3	0.54	0.657	1	0.52	0.477	3	3.32	0.036
FDiv	3	0.63	0.600	1	0.06	0.807	3	0.49	0.692
FDis	3	3.02	0.044	1	1.57	0.223	3	0.79	0.509
S	3	5.39	0.005	1	0.06	0.806	3	0.40	0.752
D	3	1.49	0.242	1	0.59	0.450	3	1.55	0.226
Q	3	6.24	0.003	1	1.04	0.318	3	0.74	0.536
R	3	4.30	0.014	1	2.21	0.149	3	0.86	0.473
Vi	3	50.01	<0.001	1	0.21	0.644	3	2.89	0.035

CWM, community-weighted means and FDis, functional dispersion for different plant traits [LA, leaf area (mm^2^); LTh, leaf thickness (mm); SLA, specific leaf area (mm^2^ mg^−1^); LDMC, leaf dry matter content (mg g^−1^); WD, wood density (g cm^−3^)]; FRic, functional richness; FEve, functional evenness; FDiv, functional divergence; FDis, functional dispersion; S, species richness; D, Gini–Simpson index; Q, Rao quadratic diversity (measure of functional diversity as well as average community-level vulnerability); R, community-level functional redundancy; Vi, Species-level vulnerability.

## Data Availability

The data presented in this study are available on request from the corresponding author.

## Supplementary Materials

The following supporting information can be downloaded at: https://www.mdpi.com/article/10.3390/plants11233284/s1, Figure S1. Location of the study zone and plots (northwest of Caquetá state, Colombian Andean–Amazonian transition). DP, degraded pasture; EF, early forest; IF, intermediate forest; OF, old-growth forest; Figure S2. Principal component analysis (PCA) with 28 above and belowground environmental parameters and the sampling plots projected on the ordination plane PC1/PC2. The color of the vectors indicates the contribution of the variables to the PCs; 95% confidence ellipses. (a) and (b), sampling plots grouped by chronosequence (DP, degraded pasture; EF, early forest; IF, intermediate forest; OF, old-growth forest or mature forest) and landscape (hill and mountain), respectively; Figure S3. Redundancy analysis (RDA) of community-weighted means (CWMs) of plant traits associated with different successional categories or landscape units, constrained by above- and below-ground environmental parameters. (a) successional categories (DP, degraded pasture; EF, early forest; IF, intermediate forest; OF, old-growth forest or mature forest; (b) landscape units. Ellipses represent the standard deviation around the centroid of each category or landscape. Figure S4. Correlogram of Pearson’s correlation coefficients between functional and above and belowground environmental variables. Significant correlations (*p* < 0.05) are represented by blue (positive) and red (negative) filled circles. Size and color intensity of the circles indicate the strength of correlation; Figure S5. Linear regressions with permutation tests for different functional diversity aspects vs. above- and below-ground environmental parameters during the secondary succession. Blue line represents the fitted model, and grey band represents the 95% confidence interval. (a) FRic vs. slope; (b) FRic vs. Ca; (c) FEve vs. slope; (d) FEve vs. CEC (e) FEve vs. EA; (f) FDiv vs. silt; (g) FDis vs. penetration resistance; (h) FDis vs. organic material. Figure S6. Linear regressions with permutation tests for functional dispersion (FDis) of individual plant traits vs. above- and below-ground environmental parameters during the secondary succession. (a) FDis.LA vs. penetration resistance; (b) FDis.LA vs. organic material; (c) FDis.LTh vs. pH; (d) FDis.SLA vs. Ca; (e) FDis.WD vs. slope; (f) FDis.WD vs. temperature; (g) FDis.WD vs. EA; (h) FDis.WD vs. P; (i) FDis.WD vs. EC; (j) FDis.WD vs. organic material. Table S1. List of the plant species and their functional vulnerability values by plot for each successional category in two landscape units. Figure S7. Linear regressions with permutation tests for different attributes functional vulnerability-related vs. above- and below-ground environmental parameters during the secondary succession. Blue line represents the fitted model and grey bands the 95% confidence interval. (a) Species richness (S) vs. precipitation; (b) S vs. conductivity electric (CE); (c) Rao quadratic diversity (Q) vs. penetration resistance; (d) Q vs. organic material; (e) Functional redundancy (R) vs. Mg; (f) R vs. penetration resistance.
