# Supplementary material for: Plant-Community Vulnerability in Highly Fragmented Landscapes Is Higher in Secondary Forests Than in Old Growth Forests in the Andean–Amazonian Transition
Source: Plants (Basel). 2022 Nov 29;11(23):3284. doi: 10.3390/plants11233284 (PMC9739964; doi:10.3390/plants11233284)
Supplement: Supplementary file 1 [file plants-11-03284-s001.zip › Supplementary Information v4.pdf]

Supplementary Materials

Higher functional diversity in secondary forests increases plant-community vulnerability in highly fragmented landscapes in the Andean-Amazonian transition

Carlos H. Rodríguez-León [1,2], Lilia L. Roa-Fuentes [3,*], Armando Sterling [2,4,*], Juan Carlos Suárez [5]

1 Doctorado en Ciencias Naturales y Desarrollo Sustentable, Facultad de Ciencias Agropecuarias, Universidad de la Amazonía, Florencia 180001, Caquetá, Colombia
2 Programa Modelos de Funcionamiento y Sostenibilidad, Instituto Amazónico de Investigaciones Científicas SINCHI, Florencia 180001, Caquetá, Colombia
3 Departamento de Ecología y Territorio, Facultad de Estudios Ambientales y Rurales, Pontifica Universidad Javeriana, Bogotá 110231, Colombia
4 Programa de Biología, Facultad de Ciencias Básicas, Universidad de la Amazonía, Florencia 180001, Caquetá, Colombia
5 Laboratorio de Ecofisiología, Centro de Investigaciones Amazónicas CIMAZ-MACAGUAL, Universidad de la Amazonía, Florencia 180001, Caquetá, Colombia
* Correspondence: lilia.roa@javeriana.edu.co (L.L.R.-F.); asterling@sinchi.org.co (A.S.)

Supplementary Figures

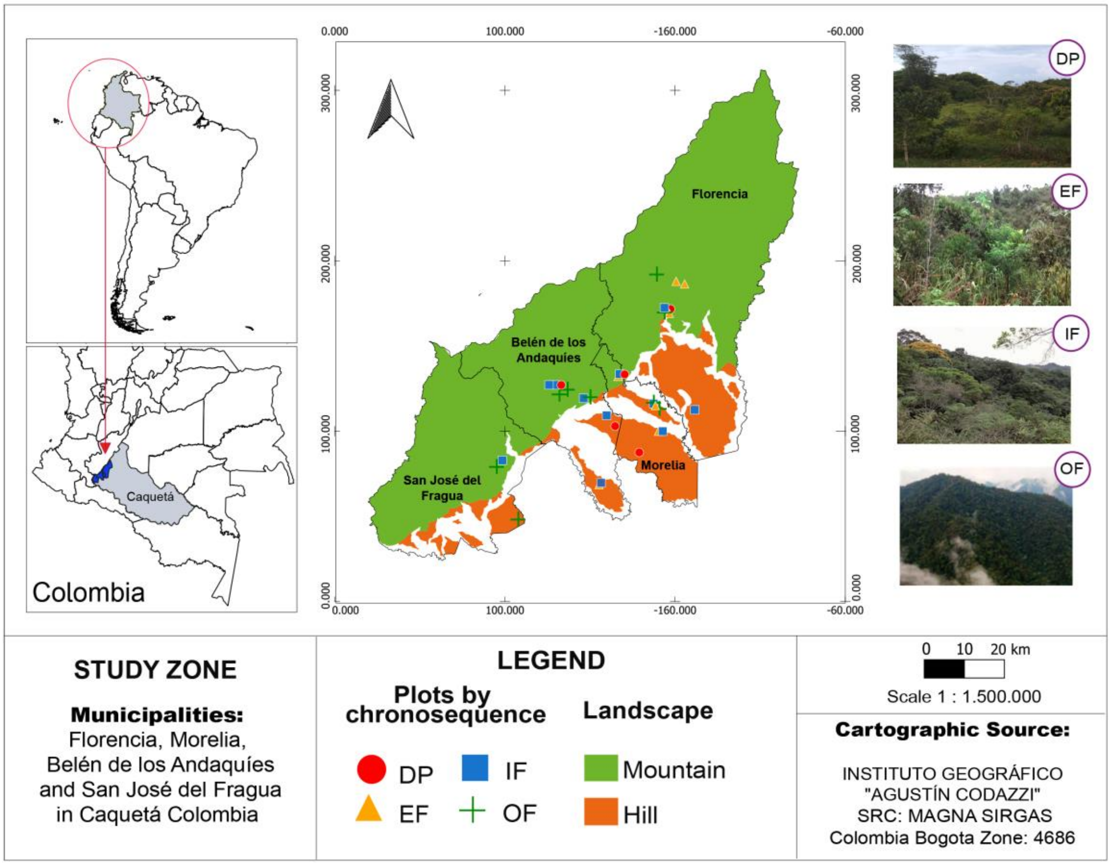

Figure S1. Location of the study zone and plots (northwest of Caquetá state, Colombian Andean-Amazonian transition). DP, degraded pasture; EF, early forest; IF, intermediate forest; OF, old-growth forest.

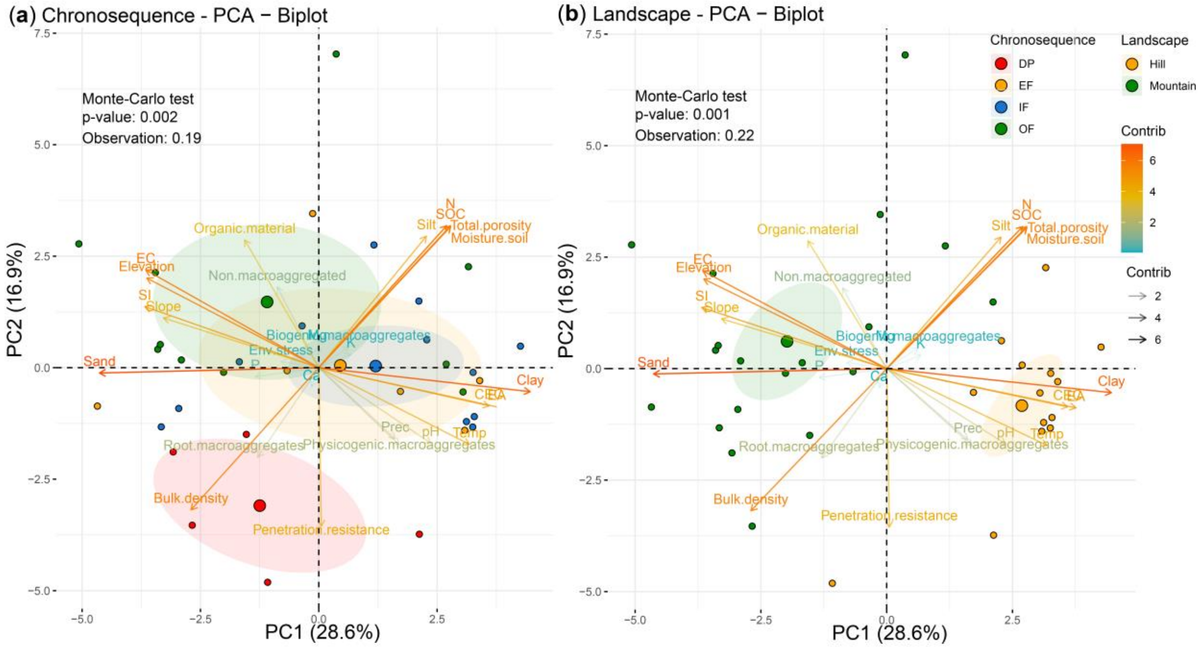

Figure S2. Principal component analysis (PCA) with 28 above and belowground environmental parameters and the sampling plots projected on the ordination plane PC1/PC2. The color of the vectors indicates the contribution of the variables to the PCs; 95% confidence ellipses. (**a**) and (**b**), sampling plots grouped by chronosequence (DP, degraded pasture; EF, early forest; IF, Intermediate forest; OF, Old-growth forest or mature forest) and landscape (Hill and mountain), respectively.

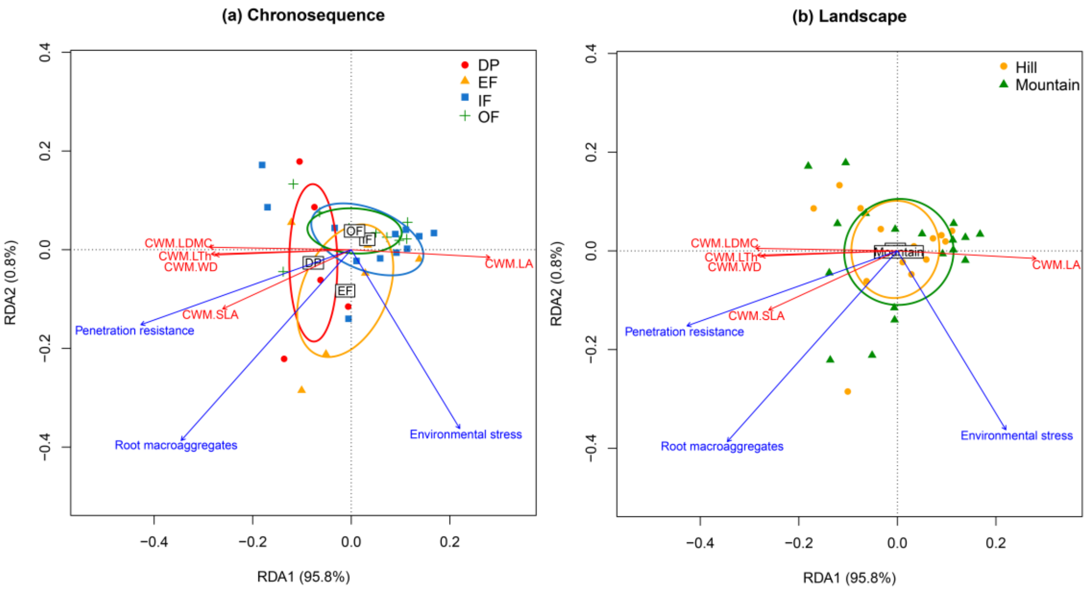

Figure S3. Redundancy analysis (RDA) of community-weighted means (CWMs) of plant traits associated with different successional categories or landscape units, constrained by above- and below-ground environmental parameters. (**a**) successional categories (DP, degraded pasture; EF, early forest; IF, Intermediate forest; OF, Old-growth forest or mature forest; (**b**) landscape units. Ellipses represent the standard deviation around the centroid of each category or landscape.

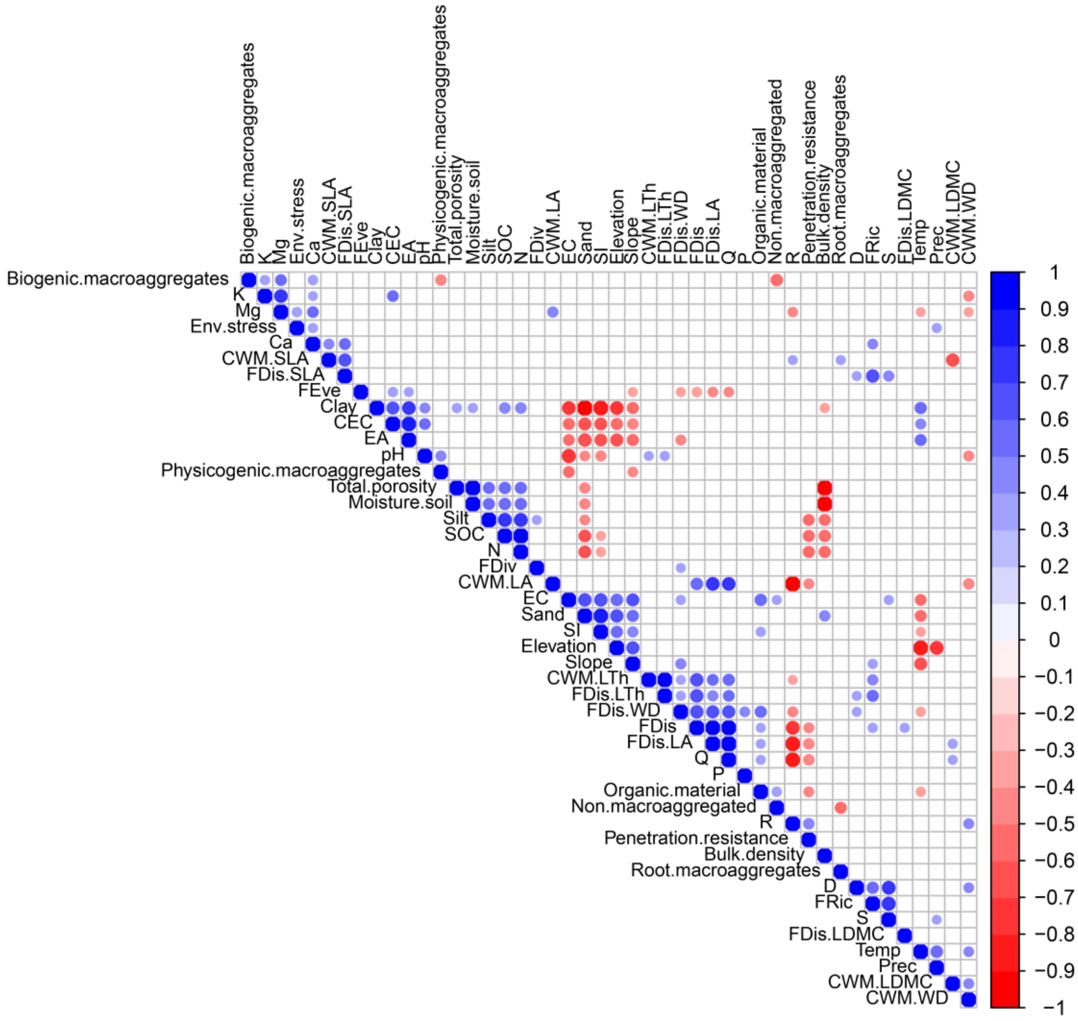

Figure S4. Correlogram of Pearson's correlation coefficients between functional and above and belowground environmental variables. Significant correlations ($p < 0.05$) are represented by blue (positive) and red (negative) filled circles. Size and color intensity of the circles indicate the strength of correlation.

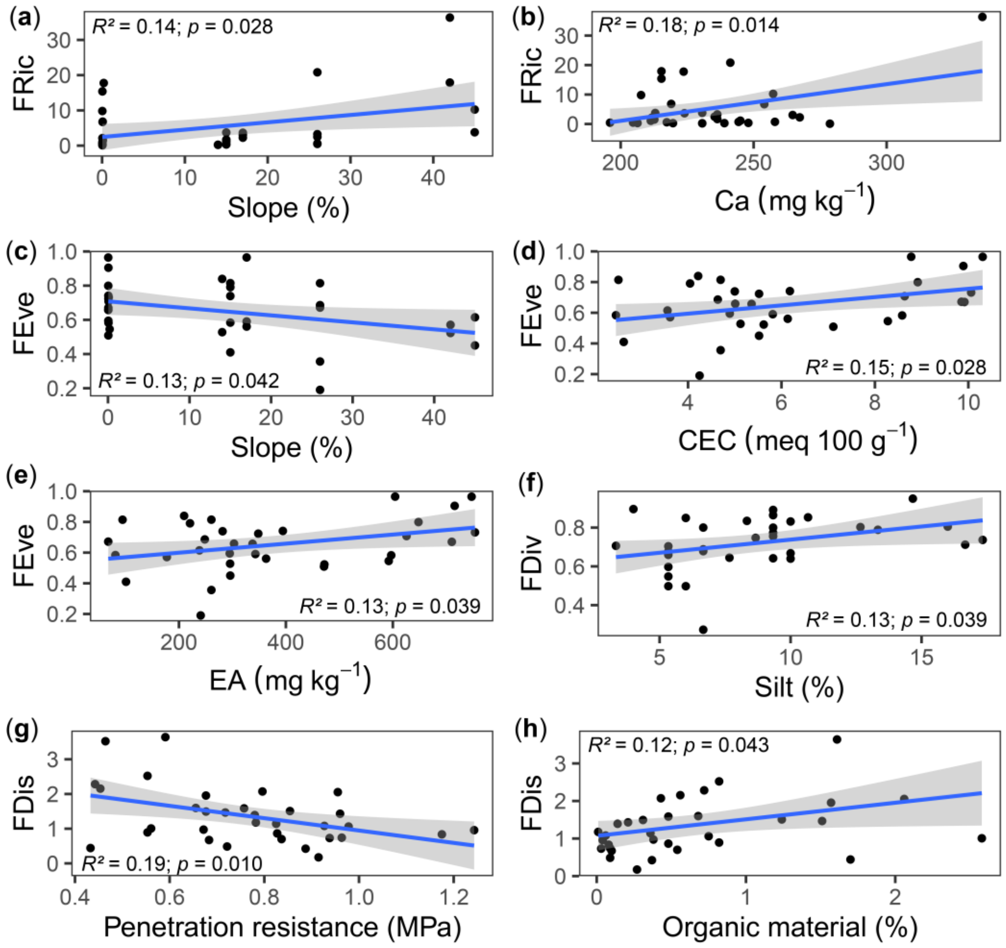

Figure S5. Linear regressions with permutation tests for different functional diversity aspects vs. above- and below-ground environmental parameters during the secondary succession. Blue line represents the fitted model, and grey band represents the 95% confidence interval. (**a**) FRic vs. slope; (**b**) FRic vs. Ca; (**c**) FEve vs. slope; (**d**) FEve vs. CEC; (**e**) FEve vs. EA; (**f**) FDiv vs. silt; (**g**) FDis vs. penetration resistance; (**h**) FDis vs. organic material.

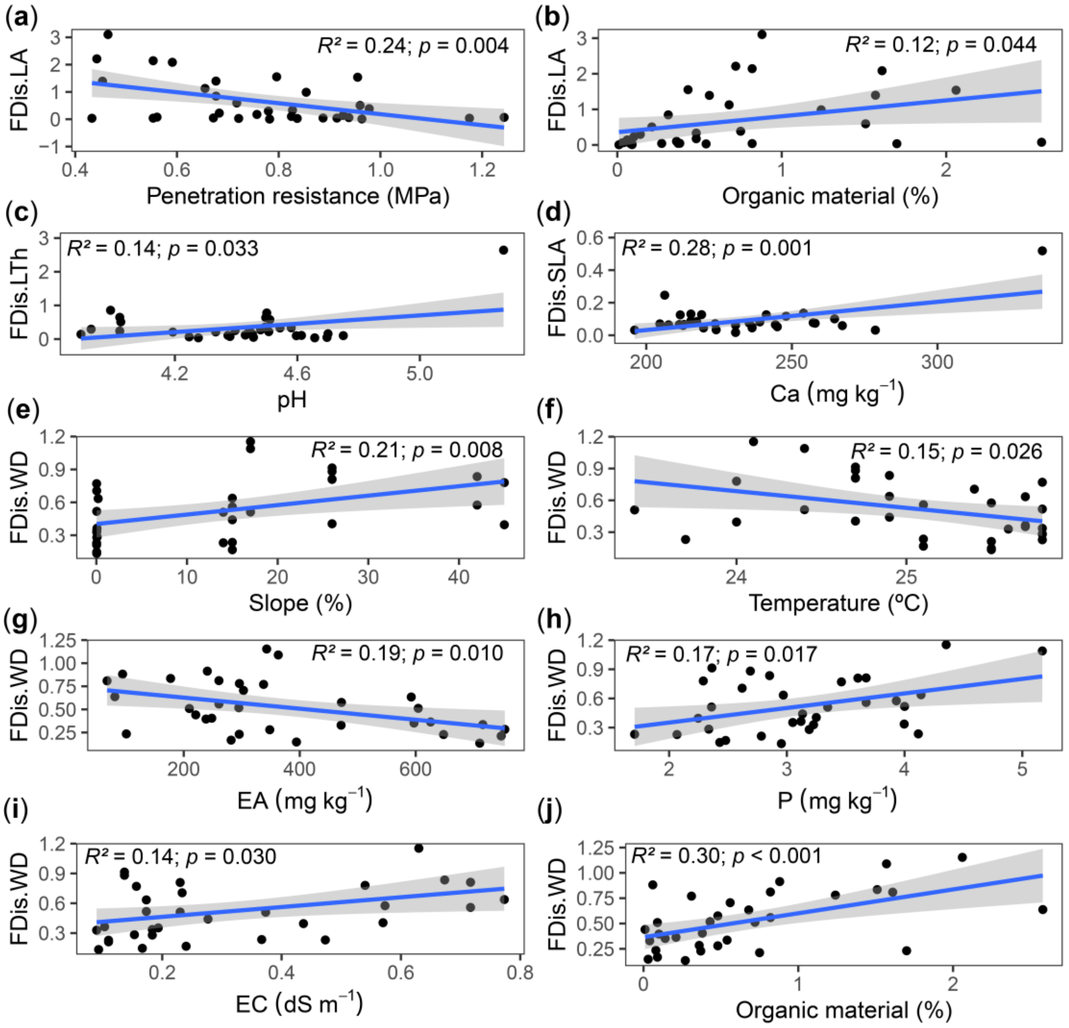

Figure S6. Linear regressions with permutation tests for functional dispersion (FDis) of individual plant traits vs. above and belowground environmental parameters during the secondary succession. (**a**) FDis.LA vs. penetration resistance; (**b**) FDis.LA vs. organic material; (**c**) FDis.LTh vs. pH; (**d**) FDis.SLA vs. Ca; (**e**) FDis.WD vs. slope; (**f**) FDis.WD vs. temperature; (**g**) FDis.WD vs. EA; (**h**) FDis.WD vs. P; (**i**) FDis.WD vs. EC; (**j**) FDis.WD vs. organic material.

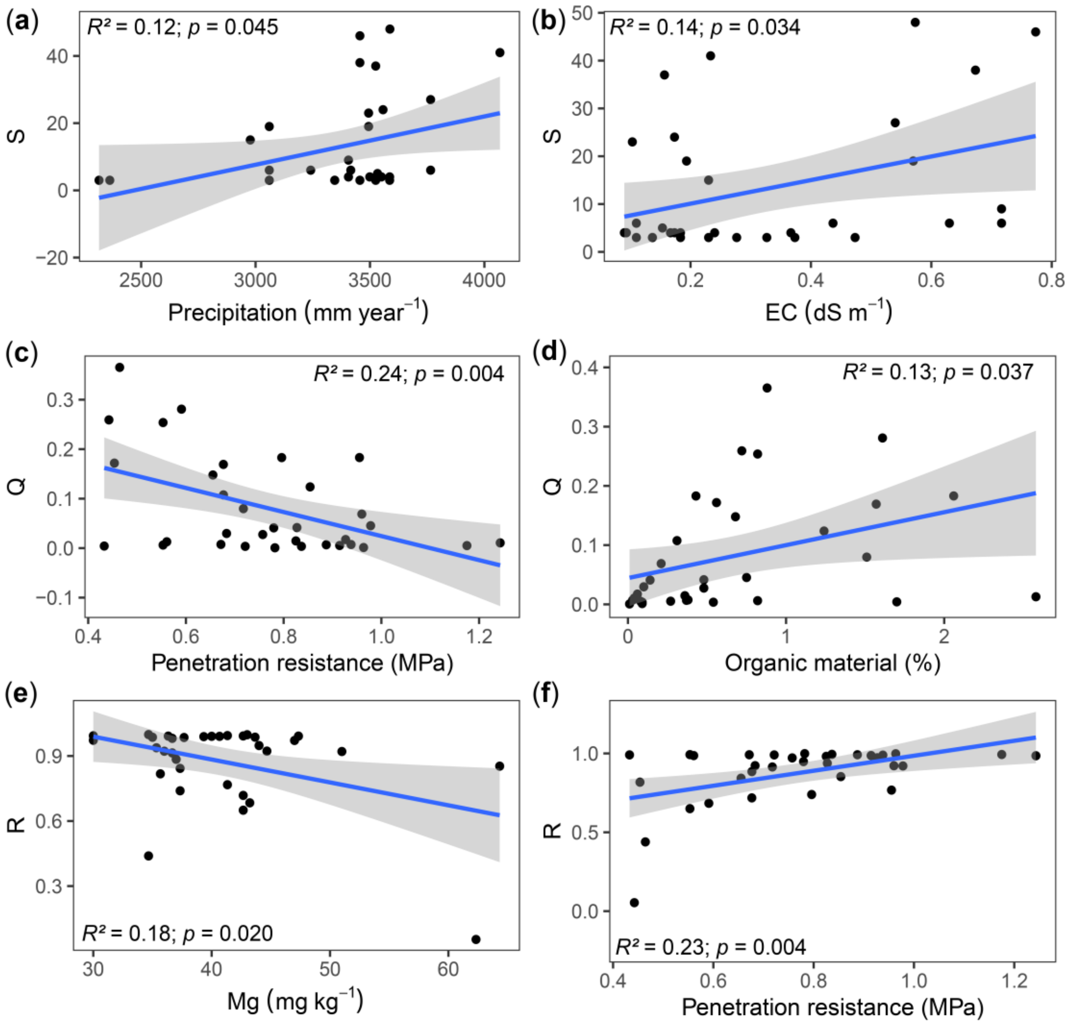

Figure S7. Linear regressions with permutation tests for different attributes functional vulnerability-related vs. above- and below-ground environmental parameters during the secondary succession. Blue line represents the fitted model and grey bands the 95% confidence interval. (**a**) Species richness (S) vs. precipitation; (**b**) S vs. conductivity electric (CE); (**c**) Rao quadratic diversity (Q) vs. penetration resistance; (**d**) Q vs. organic material; (**e**) Functional redundancy (R) vs. Mg; (**f**) R vs. penetration resistance.
